# Tailoring Type II Diabetes Treatment: Investigating the Effect of 5-HTT Polymorphisms on HbA1c Levels after Metformin Initiation

**DOI:** 10.1155/2024/7922486

**Published:** 2024-01-22

**Authors:** Taichi Ochi, Stijn de Vos, Daan Touw, Petra Denig, Talitha Feenstra, Eelko Hak

**Affiliations:** ^1^Groningen Research Institute of Pharmacy, PharmacoTherapy, Epidemiology & Economics, University of Groningen, Groningen, Netherlands; ^2^Department of Clinical Pharmacy and Pharmacology, University of Groningen, University Medical Center Groningen, Groningen, Netherlands; ^3^University of Groningen, University Medical Center Groningen, Department of Pharmacokinetics, Toxicology and Targeting, Groningen, Netherlands; ^4^Dutch National Institute for Public Health and the Environment (RIVM), Bilthoven, Netherlands

## Abstract

**Aims:**

To investigate the effect of serotonin transporter (5-HTT) polymorphisms on change in HbA1c levels six months after metformin initiation in type 2 diabetes patients.

**Materials and Methods:**

Participants of PROVALID (PROspective cohort study in patients with type 2 diabetes mellitus for VALidation of biomarkers) within the GIANTT (Groningen Initiative to ANalyse Type 2 Diabetes Treatment) cohort who initiated metformin were genotyped for combined 5-HTTLPR/rs25531 (L^∗^L^∗^, L^∗^S^∗^, and S^∗^S^∗^) and 5-HTT VNTR (STin 2.12, 12/-, and 10/-) polymorphisms, respectively. Multiple linear regression was applied to determine the change in HbA1c level from baseline date to six months across 5-HTTLPR/VNTR genotype groups, adjusted for baseline HbA1c, age, gender, triglyceride level, low-density lipoprotein level, and serum creatinine.

**Results:**

157 participants were included, of which 56.2% were male. The average age was 59.3 ± 9.23 years, and the mean baseline HbA1c was 7.49% ± 1.21%. 5-HTTLPR was characterized in 46 patients as L^∗^L^∗^, 70 patients as L^∗^S^∗^, and 41 patients as S^∗^S^∗^ genotypes. No significant association was found between 5-HTTLPR and 5-HTT VNTR genotypes and change in HbA1c after adjustments.

**Conclusions:**

5-HTT polymorphisms did not affect HbA1c levels six months after the start of metformin. Further long-term studies in large samples would be relevant to determine which polymorphisms can explain the variation in response to metformin treatment.

## 1. Introduction

As the prevalence of type 2 diabetes (T2D) increases around the world, the call for more personalised treatment grows to reduce complications and make current treatment more cost-effective [1]. In 2021, T2D affected more than 61 million people in Europe with the total healthcare expenditure totalling $189 billion [1]. Although many new classes of drugs have been developed for T2D, metformin is still widely accepted as first-line therapy in the majority of patients without high-risk comorbid conditions. However, not all T2D patients show a good response to metformin, and the response is highly variable. This can only be, in part, explained by demographic or disease factors [2].

Over the years, investigations to define genetic variations which impact metformin pharmacokinetics have led to identifying variants in glucose transporter 2 (GLUT2; *SLC2A2*) [3], ATM serine/threonine kinase (*ATM*) [4], multidrug and toxin extrusion 1 (MATE; *SLC47A1*) [5], and organic cation transport (OCT1; *SLC22A1*) [6, 7]. These transporters were observed to mediate metformin absorption in hepatocytes and impact distribution, leading to varied drug kinetics and metformin response. However, the findings have not been conclusive due to mixed results in subsequent studies [8, 9].

Whereas the above studies have focused on the transporter activity within the liver, other studies indicate that the gut also plays a role in metformin pharmacokinetic action. Four transporters in the gut have been found to be relevant in metformin transport *in vitro.* Within Caco-2 cell monolayers, a cellular model of the human intestinal epithelium, *SLC6A4* (5-HTT, serotonin transporter) accounted for 20% of metformin transport activity [10]. Further investigation on the polymorphisms of 5-HTTLPR (5-HTT linked promoter region) found that patients carrying short allele (S^∗^) polymorphisms were associated with a greater risk for gastrointestinal intolerance to metformin [11]. Furthermore, the activity of 5-HTT is modulated by variations in the intron 2 variable number of tandem repeats (STin 2 VNTR), which may impact metformin transport as well [12]. We therefore hypothesise that polymorphic regions of 5-HTT (i.e., 5-HTTLPR and 5-HTT VNTR) may be associated with HbA1c response in T2D patients after initiating metformin treatment.

Our investigation, therefore, is aimed at assessing the association of 5-HTTLPR and STin 2 VNTR polymorphisms with HbA1c levels six months after initiation of metformin treatment in T2D patients in a real-world setting.

## 2. Methods and Materials

### 2.1. Study Design and Patient Population

This cohort study comprised of patients from the GIANTT (Groningen Initiative to ANalyse Type 2 Diabetes) project participated in PROVALID (PROspective cohort study in patients with type 2 diabetes mellitus for VALidation of biomarkers) who initiated metformin [13]. Metformin initiation was defined as patients receiving a first prescription of metformin with a maximum dose of 1000 mg between January 2007 and December 2013, without a prescription for any glucose-regulating drug in the preceding year.

The GIANTT database consists of data from electronic medical records (EMRs) of primary care patients with T2D [14]. It provides prescription data, morbidity data, routine laboratory test results, and physical examinations of individual patients. In line with Dutch primary care guidelines for T2D patients, HbA1c is commonly measured at least once a year. Per the code of conduct of data usage in health research, no ethics committee approval is needed for research from anonymous medical records in the Netherlands.

**Figure 1:**
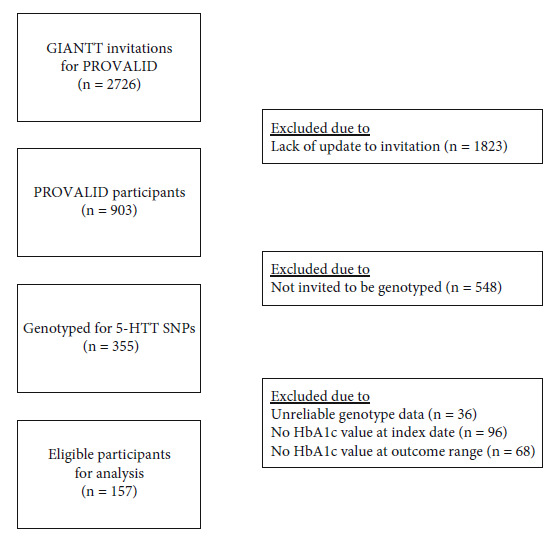
Flowchart of study subject selection. GIANTT: Groningen Initiative to ANalyse Type 2 Diabetes; PROVALID: PROspective cohort study in patients with type 2 diabetes mellitus for VALidation of biomarkers; 5-HTT: serotonin transporter; SNP: single nucleotide polymorphisms.

Funding for PROVALID was provided in part by the European Union (grant agreement: #241544, Systems Biology towards Novel Chronic Kidney Disease Diagnosis and Treatment). PROVALID was approved by the Medical Ethics Committee of the University Medical Center of Groningen (UMCG)). In total, 2726 patients were invited to participate, and written informed consent to use blood samples to determine relevant genetic information was obtained from participating patients. Consent was obtained prior to any study-specific procedure. For cost reasons, 5-HTTLPR polymorphisms were genotyped (Figure 1) in a random sample of 355 of the 903 participating patients (study approval reference #: NL35350.042.11, METC number 2011.297). Patients from this sample were excluded when the genotype data were not reliable or the HbA1c values for the outcomes were missing.

### 2.2. Genotyping

Patients were genotyped for 5-HTTLPR (long or short allele), rs25531 (A or G allele), and 5-HTT VNTR (STin2 alleles: 9, 10, or 12 repeats). DNA was extracted from blood samples from the PROVALID cohort and genotyped using the iPlex® Gold platform (Agena Bioscience GmbH, Hamburg, Germany) at the Department of Genetics, University Medical Center Groningen, the Netherlands.

The 5-HTTLPR polymorphism is categorised by long (L) and short (S) variants within the SERT gene (*SLC6A4*) promoter region, with the S variant linked with decreased SERT expression and function. rs25531 is located within the 5-HTTLPR region and further modulates SERT expression in long variants of 5-HTTLPR through allelic combination. L_A_ carriers are found to have higher SERT expression while in L_G_ lower SERT expression similar to that in S allele carriers was found [11]. For the analysis, the triallelic 5-HTTLPR genotypes were grouped as L^∗^L^∗^ (L_A_L_A_), L^∗^S^∗^(L_A_L_G_ and L_A_S), and S^∗^S^∗^ (SS, L_G_S, and L_G_L_G_). 5-HTT VNTR STin2 polymorphisms, also known as intron 2 VNTR, were categorised by 9-repeat alleles, 10-repeat alleles, and 12-repeat alleles. STin2.12 has been associated with increased enhancer properties for SERT expression compared to STin2.10 and 2.9 [15]. The 5-HTT VNTR STin2 genotypes were combined into two groups of normal and low function as STin2.12/– (12/12, 12/10, and 12/9) and STin2.10/– (10/10, 10/9, and 9/9) [16].

### 2.3. Outcome Variable

**Figure 2:**
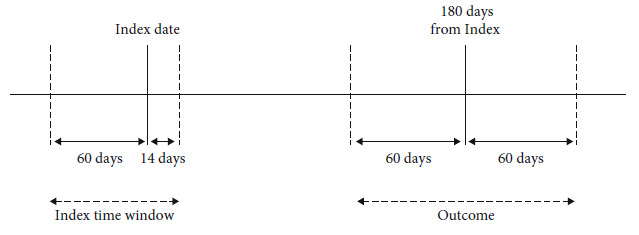
Observational cohort study design.

The primary outcome is the change in HbA1c level from baseline to 6 months follow-up. For HbA1c at 6 months follow-up, a predefined 60-day time window around 180 days after metformin initiation (baseline date, Figure 2) was used. In case of multiple measurements, the measurement nearest to the 180 days was selected from the GIANTT database. That is, the 6 months follow-up outcome measure will be taken six months after the baseline date, or at the nearest date to six months within 60 days. The change in HbA1c over the course of the study period was determined by the difference between the baseline measurement and the six-month measurement.

### 2.4. Baseline Characteristics

Baseline clinical covariates that may influence the association between the 5-HTT polymorphisms and the primary outcome included HbA1c at baseline, age, gender, body mass index (BMI), systolic blood pressure, low-density lipoprotein levels, total cholesterol, high-density lipoprotein levels, triglyceride levels, and serum creatinine levels. Age, blood pressures, lipid levels, and serum creatinine levels at baseline were defined as those measured within a time window of 60 days prior to and up to 14 days after the baseline date. The baseline measurements will be the measurement nearest to the baseline date within this time window. BMI was obtained from the most recent measurement documented in the year before the baseline date or calculated from the most recent weight in the year before and height in the 5 years before the baseline date.

### 2.5. Missing Data for Covariates

For sensitivity analyses, multiple imputation using a chained equation (MICE) was applied to impute missing covariate information, and, separately, the complete case analysis was conducted. After checking missing data patterns, thirty dataset replicates were imputed based on the highest percentage of missing data in the variables (BMI with 26.1% of data missing). Imputed data were reviewed by fit and inspection comparing the means of the 30 imputed datasets to the original data using ANOVA. After fitting the multiple linear regression model in each of the 30 imputed datasets, the coefficients were averaged using Rubin's rule.

### 2.6. Statistical Analysis

Patient characteristics were summarised using descriptive statistics, stratified per 5-HTTLPR and 5-HTT VNTR STin2 genotypes. ANOVA was conducted to test for statistical differences in these characteristics between the genotype groups. Chi-squared test was conducted to determine the independence between 5-HTTLPR and 5-HTT VNTR variants (Supplementary Table 2).

Multiple linear regression was conducted to determine whether the 5-HTT genotypes (5-HTTLPR and STin2 VNTR) were associated with the change in HbA1c level over six months after metformin initiation. The regression was adjusted for age, sex, baseline HbA1c, systolic blood pressure, triglyceride level, low-density lipoprotein level, and serum creatinine levels. Baseline total cholesterol and high-density lipoprotein were not included in the analyses as the collinearity with low-density lipoprotein level was high (VIF: >8). Statistical analysis was conducted with SPSS software (release 26.0). The statistical significance level was *p* value < 0.05.

## 3. Results

The study analysed data from 157 participants, 88 men and 69 women, with an average age of 59.3 ± 9.3 years and baseline HbA1c of 7.49 ± 1.21% (Supplementary Table 1). 5-HTTLPR was characterized in 46 patients as L^∗^L^∗^, 70 patients as L^∗^S^∗^, and 41 patients as S^∗^S^∗^ genotype (Table 1). HbA1c after six months between 5-HTTLPR were found to be 6.6 ± 0.5, 6.6 ± 0.6, and 6.6 ± 0.6 (%) for L^∗^L^∗^, L^∗^S^∗^, and S^∗^S^∗^, respectively. 5-HTT VNTR was characterized in 126 patients as STin 2.12/- and in 31 patients as STin 2.10/-. No significant difference in baseline characteristics was found between the 5-HTT genotypes for HbA1c at baseline and after six months when comparing the groups with ANOVA.

Within the main multiple linear regression analysis, no significant association was found between 5-HTTLPR and 5-HTT VNTR genotypes and change in HbA1c (Table 2). HbA1c at baseline was found to be significant as a covariate in the regression analyses (*B* = 0.66, *p* = 0.01). In the sensitivity analysis with the imputed dataset, no relevant differences were found between the baseline characteristics when compared to the main analysis. Furthermore, no associations were found between 5-HTTLPR and 5-HTT VNTR genotypes and changes in HbA1c. For the complete case analysis, 89 patients were included. Again, no significant differences were found between the baseline characteristics, nor any associations of the 5-HTT genotypes with change in HbA1c.

## 4. Discussion

Our investigation focused on primary care T2D patients to estimate the short-term effect of 5-HTT polymorphisms on HbA1c levels after the start of metformin treatment. No significant associations were found between the 5-HTT genotypes and change in HbA1c level over six months after metformin initiation.

While STin2 was not found to impact HbA1c response after metformin initiation in our cohort, its polymorphisms are known to affect 5-HTT expression [12]. We found 5-HTTLPR and STin2 VNTR to be independent of each other, but how the 5-HTT activity was modulated by these different polymorphisms could not be determined in this investigation. Therefore, whether 5-HTT polymorphisms impact metformin activity between the stratified patients is yet to be clearly defined. It may be possible that a larger cohort may provide further clarification on whether the STin2 VNTR genotypes modulate 5-HTT activity, in conjunction with 5-HTTLPR. As in this study, the stratification of STin2 may have proved not specific enough due to the categorisation of STin2.12, 2.10, and 2.9 in the analysis.

Our study looked at a period of six months after metformin initiation, looking to investigate the study period in which 5-HTTLPR was found to play a role in metformin intolerance [11]. However, most T2D patients may take metformin for several years. It has been argued that the longitudinal disease progression of T2D in patients taking metformin should be taken into account [17]. Using a longer follow-up, their results provide an indication of the utility of incorporating diseased progression when determining genetic variants that may impact how a patient's response to treatment [17]. The study did not indicate the list of genes that were included; therefore, it is not possible to ascertain whether 5-HTT (*SLC6A4)* polymorphisms were investigated. Therefore, while our results were inconclusive for the time window of six months, extending the investigation to a greater time window incorporating disease progression may provide additional insight on how to utilise genetic information to manage metformin treatment.

Whether 5-HTT polymorphisms impact metformin activity may likely also be dependent on the interaction with other genes of interest. This study builds on an investigation where OCT1 (*SLC22A1*) was investigated and found to play a role in the uptake of metformin [18]. However, as this data was not available, it was not able to be replicated here. Furthermore, renal function and active kidney transport are involved in drug kinetic variability, which this study did not investigate further. Additional investigations with the other genes of interest could not be conducted in our study as the genetic data was not available.

Patients usually initiate metformin at doses of not more than 1000 mg per day [2]. This dose may be increased when insufficient glucose-lowering effect is seen. However, some patients may not tolerate higher dosing of metformin which could in part explain variability in HbA1c response [19]. Unfortunately, we did not have data on intolerance, so we cannot explore the impact of intolerance on our findings.

### 4.1. Strengths and Limitations

The study was conducted in a real-world cohort of patients with T2D. Our study population stems from a representative cohort of Dutch primary care patients with T2D. Patients with T2D in the Netherlands generally start metformin dosage at 500 mg and increase as treatment duration is prolonged. Due to the short duration of the study, there were no changes in medication dosage. However, as this study represents a subset of the initial cohort when compared to the demographics of T2D patients in the Netherlands, our study group is on average younger [20]. From the 355 patients that were genotyped, 157 could be included in the main analysis due to missing HbA1c values. This was partly due to the fact that in some patients, HbA1c values were not measured before the start of treatment. For other patients, no HbA1c value was available within the set time window for the outcome. The final analysis was conducted only in participants whose data fit in the time window to prevent misclassification of the outcome. To test the effect of missing data in covariates, we conducted multiple imputations, which did not change our findings. It would have an option to follow up with the healthcare practitioners and participants regarding the missing data. However, due to the long period of time passing since the data was collected, the risk of recall bias would be very high.

This resulted in a relatively small study cohort. A larger cohort would enable to individually study each specific genotype within 5-HTT VNTR and provide further insight into the effect each genotype has regarding response to metformin. However, as the literature is not clear regarding 5-HTT VNTR subtypes, rather than looking to categorise each group, focusing on 5-HTTLPR genotypes may prove more fruitful for future studies.

## 5. Conclusion

This study indicates that 5-HTT genotyping does not contribute to explaining variation in HbA1c response six months after metformin initiation in T2D patients. To improve the personalised treatment of T2D patients, it is recommended that future investigations investigate longer time periods in large samples, including also the impact of disease progression and metformin intolerance. This would further elucidate whether genetic testing would help optimise treatment for long-term metformin use.

## Figures and Tables

**Table 1:** Participant characteristics—split between 5-HTTLPR and 5-HTT VNTR genotypes (*n* = 157).

Characteristics	5-HTTLPR				5-HTT VNTR		
Genotype breakdown	L^∗^L^∗^ (*n* = 46)	L^∗^S^∗^ (*n* = 70)	S^∗^S^∗^ (*n* = 41)	Sig	STin 2.12/- (*n* = 126)	STin 2.10/- (*n* = 31)	Sig
Total number of participants (%)							
Male	31 (67.4%)	35 (50%)	22 (53.7%)		75 (59.5%)	13 (41.9%)	
Female	15 (32.6%)	35 (50%)	19 (46.3%)		51 (40.5%)	18 (58.1%)	
Age in years (mean ± SD)	60.20 ± 8.79	58.74 ± 7.72	59.59 ± 10.98	0.63	58.98 ± 9.22	50.74 ± 7.81	0.67
Baseline HbA1c (%)	7.42 ± 0.96	7.53 ± 1.34	7.77 ± 1.34	0.42	7.53 ± 1.22	7.67 ± 1.36	0.59
HbA1c value 6 months (%)	6.59 ± 0.47	6.58 ± 0.61	6.60 ± 0.56	0.85	6.58 ± 0.58	6.63 ± 0.47	0.06
Baseline BMI	30.54 ± 3.90	32.41 ± 5.34	30.16 ± 5.68	0.68	31.09 ± 5.21	31.89 ± 4.89	0.99
Baseline blood pressure (mmHg)							
Systolic	143.39 ± 18.7	140.21 ± 15.66	146.40 ± 19.48	0.25	141.72 ± 17.29	147.44 ± 18.98	0.15
Diastolic	85.61 ± 11.73	83.93 ± 9.25	83.65 ± 10.81	0.67	83.91 ± 9.74	86.27 ± 12.89	0.32
Baseline lipid levels (mmol/L)							
Total cholesterol	5.04 ± 1.03	5.26 ± 1.44	4.97 ± 1.29	0.52	5.05 ± 1.18	5.39 ± 1.68	0.23
HDL cholesterol	1.16 ± 0.31	1.25 ± 0.31	1.22 ± 0.36	0.49	1.21 ± 0.34	1.25 ± 0.28	0.51
Triglycerides	2.26 ± 1.04	1.96 ± 0.87	1.92 ± 1.08	0.27	2.02 ± 0.94	2.07 ± 1.13	0.81
LDL cholesterol	2.90 ± 0.96	3.16 ± 0.98	2.98 ± 0.93	0.40	3.05 ± 0.98	3.04 ± 0.88	0.96
Serum creatinine levels (mmol/L)	81.29 ± 12.86	80.31 ± 16.99	80.58 ± 17.04	0.95	81.05 ± 16.23	79.15 ± 13.66	0.59

Sig: significance; HbA1c: hemoglobin A1c; HDL: high-density lipoprotein; LDL: low-density lipoprotein; 5-HTTLPR: serotonin transporter linked promoter region; 5-HTT VNTR: serotonin transporter variable number tandem repeat in the second intron.

**Table 2:** Multiple linear regression for the difference in HbA1c between baseline and six months.

Baseline predictors	Standard analysis (*n* = 157)			Imputed analysis (*n* = 157)			Complete case (*n* = 89)		
	*B*	95.0% CI for *B*	*p* value	*B*	95.0% CI for *B*	*p* value	*B*	95.0% CI for *B*	*p* value
Age	-0.01	-0.03–0.01	0.12	-0.004	-0.02–0.01	0.44	-0.01	-0.03–0.01	0.12
Sex	-0.05	-0.28–0.18	0.68	-0.01	-0.22–0.2	0.93	-0.05	-0.28–0.18	0.68
5-HTTLPR (L^∗^L^∗^)									
L^∗^S^∗^	-0.01	-0.26–0.24	0.94	-0.03	-0.25–0.2	0.81	-0.01	-0.26–0.24	0.94
S^∗^S^∗^	-0.05	-0.34–0.24	0.74	-0.01	-0.26–0.25	0.97	-0.05	-0.34–0.24	0.74
5-HTT VNTR (12/-)									
10/-	0.15	-0.13–0.43	0.29	-0.03	-0.27–0.21	0.79	0.15	-0.13–0.43	0.29
HbA1c at baseline	0.66	0.53–0.79	0.01^∗^	0.89	0.82–0.97	<0.001^∗∗^	0.66	0.53–0.79	<0.001^∗∗^
BMI (up to baseline)	0.004	-0.02–0.03	0.71	0.01	-0.02–0.04	0.34	0.004	-0.02–0.03	0.71
Systolic blood pressure (up to baseline)	-0.004	-0.01–0.01	0.24	-0.001	-0.01–0.01	0.95	-0.004	-0.01–0.01	0.24
Triglycerides (up to baseline)	-0.07	-0.17–0.04	0.20	-0.08	-0.19–0.02	0.12	-0.07	-0.17–0.04	0.20
LDL (up to baseline)	0.08	-0.05–0.2	0.22	0.003	-0.11–0.11	0.96	0.08	-0.05–0.2	0.22
Serum creatinine (up to baseline)	-0.001	-0.01–0.01	0.97	0.001	-0.01–0.01	0.70	-0.001	-0.01–0.01	0.97

HbA1c: hemoglobin A1c; HDL: high-density lipoprotein; LDL: low-density lipoprotein; 5-HTTLPR: serotonin transporter linked promoter region; 5-HTT VNTR: serotonin transporter variable number tandem repeat in the second intron; ^∗^*p* < 0.05; ^∗∗^*p* < 0.001.

## Data Availability

Data is available on request due to privacy/ethical restrictions.

## Supplementary Materials

Supplementary Materials Supplementary 1: patient characteristics (total). Supplementary 2: cross-tabulation and chi-squared test of 5-HTTLPR and VNTR genotypes.
