# Supplementary material for: Tailoring Type II Diabetes Treatment: Investigating the Effect of 5-HTT Polymorphisms on HbA1c Levels after Metformin Initiation
Source: J Diabetes Res. 2024 Jan 22;2024:7922486. doi: 10.1155/2024/7922486 (PMC10824573; doi:10.1155/2024/7922486)
Supplement: Supplementary Materials — Supplementary 1: patient characteristics (total). Supplementary 2: cross-tabulation and chi-squared test of 5-HTTLPR and VNTR genotypes. [file 7922486.f1.docx]

**Supplementary Table 1 – Patient Characteristics (Total)**

| **Characteristics** | | **(n = 157)** | **Missing (%)** |
| --- | --- | --- | --- |
| Total number of participants (%) | |  |  |
|  | Male | 78 (56.1%) |  |
|  | Female | 61 (43.9%) |  |
| Age in years (Mean ± SD) | | 59.33 ± 9.26 |  |
|  | Baseline HbA1c (%) | 7.49 ± 1.21 |  |
|  | HbA1c value 6 months (%) | 6.56 ± 0.52 |  |
|  | Baseline BMI | 30.99 ± 5.51 | 41 (26.1%) |
| Baseline blood pressure (mmHg) | |  |  |
|  | Systolic | 142.32 ± 17.65 | 25 (15.9%) |
|  | Diastolic | 84.07 ± 10.42 | 25 (15.9%) |
| Baseline lipid levels (mmol/L) | |  |  |
|  | Total Cholesterol | 5.03 ± 1.16 | 29 (18.5%) |
|  | HDL Cholesterol | 1.20 ± 0.31 | 30 (19.1%) |
|  | Triglycerides | 2.05 ± 0.96 | 33 (21%) |
|  | LDL Cholesterol | 2.99 ± 0.94 | 31 (19.7%) |
| Serum Creatinine levels (mmol/L) | | 80.92 ± 16.33 | 14 (15.3%) |
| Antidepressant use (n) | | 17 (10.8%) |  |

HbA1c, hemoglobin A1c. HDL, high density lipoprotein. LDL, low density lipoprotein.

**Supplementary Table 2 – Cross-Tabulation and Chi-Squared Test of 5-HTTLPR and VNTR genotypes**

|  |  | **STin2 12/-** | **STin2 10/-** | **Total** |
| --- | --- | --- | --- | --- |
| **5-HTTLPR** | Count | 31 | 15 | 46 |
| **L*L*** | *Expected Count* | 36.9 | 9.1 | 46 |
|  | Count | 57 | 13 | 70 |
| **L*S*** | *Expected Count* | 56.2 | 13.8 | 70 |
|  | Count | 38 | 3 | 41 |
| **S*S*** | *Expected Count* | 32.9 | 8.1 | 41 |
| **Total** | Count | 126 | 31 | 157 |
|  | *Expected Count* | 126 | 31 | 157 |

5-HTTLPR, Serotonin Transporter Linked Promoter Region. STin2, Serotonin Transporter Variable Number Tandem Repeat in the Second Intron.
